# miR-324-3p suppresses migration and invasion by targeting WNT2B in nasopharyngeal carcinoma

**DOI:** 10.1186/s12935-016-0372-8

**Published:** 2017-01-03

**Authors:** Chao Liu, Guo Li, Nianting Yang, Zhongwu Su, Shuiting Zhang, Tengbo Deng, Shuling Ren, Shanhong Lu, Yongquan Tian, Yong Liu, Yuanzheng Qiu

**Affiliations:** 1 Department of Otolaryngology Head and Neck Surgery, Xiangya Hospital, Central South University, 87 Xiangya Road, Changsha, 410008 Hunan China; 2 Otolaryngology Major Disease Research Key Laboratory of Hunan Province, Changsha, 410008 Hunan China

**Keywords:** Nasopharyngeal carcinoma, miR-324-3p, WNT2B, Invasion

## Abstract

**Background:**

Nasopharyngeal carcinoma (NPC) is a malignant epithelial carcinoma of the head and neck with strong ability of invasion and metastasis. Our previous study indicated that miR-324-3p, as a tumor-suppressive factor, could regulate radioresistance of NPC cells by targeting *WNT2B*. The purpose of this study is to investigate the role of miR-324-3p on migration and invasion in NPC cells.

**Methods:**

Quantitative real time PCR was applied to measure the expression level of miR-324-3p and *WNT2B* mRNA in both cells and tissues, and the expression level of *WNT2B* protein was determined by western blotting. The capacity of migration and invasion were tested by using wound healing and transwell invasion assay.

**Results:**

Ectopic expression of miR-324-3p or silencing its target gene *WNT2B* could dramatically suppress migration and invasion capacity of NPC cells. Meanwhile, the alterations of miR-324-3p in NPC cells could influence the expression level of the biomarkers of epithelial-mesenchymal transition (EMT), including E-cadherin and Vimentin. Moreover, the expression of miR-324-3p was obviously downregulated and *WNT2B* was significantly upregulated in NPC tissues. The expression levels of miR-324-3p and *WNT2B* were closely correlated with T stage, clinic stage and cervical lymph node metastasis of NPC (*P* < 0.05).

**Conclusion:**

miR-324-3p could suppress the migration and invasion of NPC by targeting *WNT2B* and the miR-324-3p/WNT2B pathway possibly provide new potential therapeutic clues for NPC.

**Electronic supplementary material:**

The online version of this article (doi:10.1186/s12935-016-0372-8) contains supplementary material, which is available to authorized users.

## Background

Nasopharyngeal carcinoma (NPC) is an Epstein-Barr virus associated cancer that mostly occurs in Southern China and South-Eastern Asia [1]. Despite great improvements in chemoradiotherapy over the past few decades, the overall 5-year survival rate for NPC still remains poor [2]. Strong ability to migrate and invade is a leading cause for the dismay prognosis of advanced NPC patients [3]. For this reason, exploring the molecular mechanisms underlying NPC migration and invasion is essential for the development of novel therapeutic strategies.

microRNAs (miRNAs) are a kind of small non-protein-coding RNA, which consist of 19–25 nucleotides [4]. They can inhibit target gene expression at the post-transcriptional level by pairing with the 3′ untranslated regions (3′ UTRs), 5′ UTRs or coding region of mRNAs [5, 6]. Accumulated evidences prove that dysregulated miRNAs can function as oncogenes or tumour suppressors in the initiation and progression of various human cancers [7]. In NPC, miRNAs are also reported to play important regulatory roles in many critical biological processes including cell proliferation, apoptosis, radiosensitivity and chemosensitivity [8–10]. As to migration and invasion of NPC, a series of miRNAs like miR-145 or miR-744 can inhibit or enhance NPC migration and invasion by targeting *SMAD3* or *ARHGAP5,* respectively [11, 12]. These above findings indicated that miRNAs provide a new perspective for the investigation of migration and invasion in NPC.

In our preliminary study, we have found that miR-324-3p could regulate the radioresistance of NPC cells and further confirmed *WNT2B* was the target gene of miR-324-3p [13]. Here, we focused on the roles of miR-324-3p involved in NPC migration and invasion. Our results showed that miR-324-3p inhibited migration and invasion of NPC cells and affected epithelial-mesenchymal transition (EMT) biomolecules, and the target gene *WNT2B* could enhance the migration and invasion ability of NPC. Currently, in NPC tissue specimens, miR-324-3p was found to be downregulated while *WNT2B* was upregulated. Moreover, the expression levels of miR-324-3p and *WNT2B* were associated with stages of NPC, as well as with lymph node metastasis. These results provide valuable clues toward understanding the molecular mechanisms of NPC migration and invasion.

## Methods

### Cell cultures

NPC cell lines 5-8F and 6-10B were purchased from the Cell Center of Central South University, Changsha, China. The cells were cultured in RPMI medium 1640 (Hyclone, Logan, UT, USA) with 10% foetal bovine serum (Gibco BRL, Gaithersburg, MD, USA) and were incubated at 37 °C in a humidified chamber with 5% CO_2_. Cells in an exponential growth state were used for subsequent experiments.

### Patients and tissue preparation

Primary NPC (n = 39) and normal nasopharyngeal epithelium (NPE) (n = 21) tissues were obtained from the the Department of Otolaryngology Head and Neck Surgery, Xiangya Hospital, Central South University, Changsha, China. All patients had no history of previous malignancies. Staging was performed by the 2008 NPC staging system of China. The study protocol was approved by the Research Ethics Committee of the Central South University, Changsha, China. Informed consents were obtained from all of the patients.

### Oligonucleotides and transfection

miR-324-3p mimics, WNT2B siRNA and negative control (NC) were chemically synthesized from Gene-Pharma Co., Shanghai, China. The NPC cells 5-8F and 6-10B were transfected with Lipofectamine 2000 reagent (Invitrogen, Burlington, ON, Canada) according to the manufacturer’s instructions. The transfection efficiency was observed by fluorescence microscopy, and the expression level of miR-324-3p and WNT2B were evaluated using the qRT-PCR examination System (Bio-Rad, Hercules, CA, USA).

### RNA extraction and quantitative real-time PCR (qRT-PCR) analyses

TRIzol reagent (Invitrogen, Carlsbad, CA, USA) was used to extract the total RNA from NPC cells and tissues. The All-in-One™ miRNA qRT-PCR Detection Kit (GeneCopoeia Inc., MD, USA) was applied in reverse transcription and quantitative detection of miRNAs according to the user manual. The detection of mRNAs was carried out with TaqMan Reverse Transcription Reagents and SYBR Green PCR Master Mix (Applied Biosystems, CA, USA). PCR quantification was conducted using the 2^−ΔΔCT^ method and normalized to U6 for miRNA or GAPDH for mRNA. The sequences of the primers used for the PCR are as follows: WNT2B forward, 5′-TGG CGT GCA CTC TCA GAT TT-3′ and reverse, 5′-GAC AAG ATC AGT CCG GGT GG-3′; GAPDH forward, 5′-TCC AAA ATC AAG TGG GGC GA-3′, and reverse, 5′-AGT AGA GGC AGG GAT GAT GT-3′. The technical documentation of qRT-PCR was listed in Additional file 1, and representative data of standard and melt curves of the premirs were listed in Additional file 2: Figure S1. And the validation of the stability of the reference genes between NPC and NPE was showed in Additional file 2: Figure S2.

### Wound healing assay

Wound-healing assay was used to examine the cell migration activity. When the cells were grown to reach almost total confluence (nearly 36–48 h after transfection), a 10 μl plastic pipette tip was adopted to creat an artificial wound. Then the cells were cultured in serum-free medium. The initial gap length (0 h) and the residual gap length 24–72 h after scratching were observed under the inverted microscope. Experiments were performed in triplicate.

### Cell invasion assay

A transwell invasion assay was performed according to the operating instruction. Briefly, 1 × 10^4^ cells with serum-free medium were seeded to the top chamber with Matrigel-coated membrane (BD Biosciences, Bedford, MA, USA). After a 48 h incubation period, non-invading cells on the surface of the upper chamber were removed with a cotton swab. Cells on the lower side of the chamber were fixed in paraformaldehyde, stained with crystal violet, and then counted in five random fields under microscope. Each experiment was done three times.

### Western blotting analysis

Western blotting was carried out as described previously. Total cell or tissue proteins were extracted and separated on 10% SDS-PAGE gels, and electroblotted onto PVDF (polyvinylidene fluoride) membranes (Millipore, Billerica, MA, USA). Then, the protein expression was detected by incubation with the relevant primary antibody followed by an appropriate secondary antibody. The primary antibody in current study included anti-WNT2B (1:800, Boiss Inc., Woburn, MA, USA), anti-E-cadherin and anti-Vimentin (1:800, Cell Signaling Technology, Danvers, MA, USA). Anti-β-Actin or anti-GAPDH (1:1000, Beyotime, Shanghai, China) were used as the loading control. The relative expression of WNT2B protein was calculated by the image intensity of the ratio of WNT2B and β-Actin.

### Statistical analysis

All data shown are representative results of at least three independent experiments and were expressed as mean ± standard deviation (SD). Statistical analyses were performed using a two-sided unpaired Student’s *t* test (for equal variances) or Mann–Whitney U test (for unequal variances) with SPSS 18.0 software. *P* < 0.05 was considered statistically significant.

## Results

### Ectopic expression of miR-324-3p inhibits migration and invasion of NPC cells

**Fig. 1:**
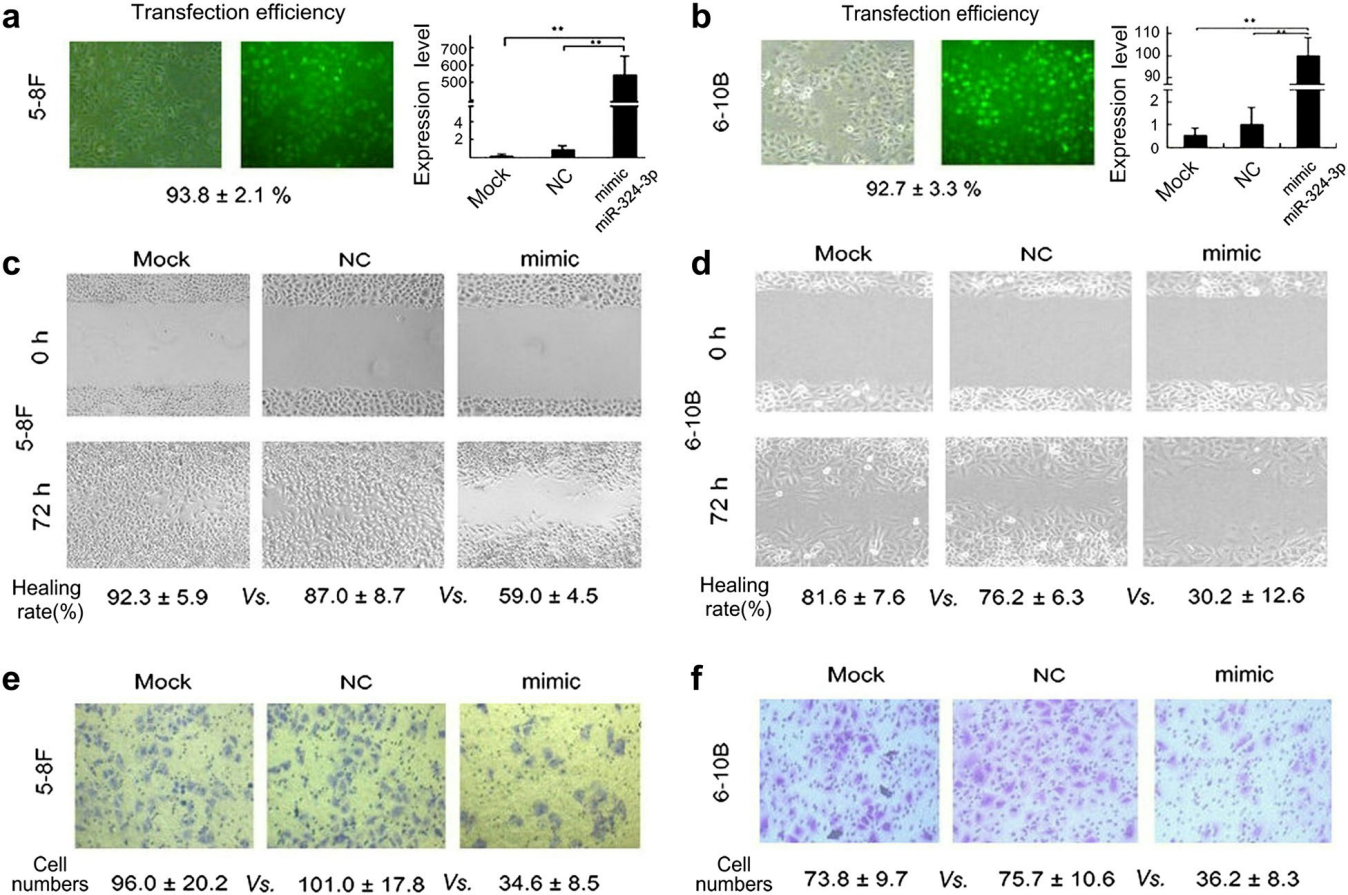
Ectopic expression of miR-324-3p inhibits migration and invasion of NPC cells. The transfection efficiency and expression of miR-324-3p were determined under fluorescent microscope and qRT-PCR in 5-8F (**a**) and 6-10B (**b**) cells. The migration of different cell groups was examined by wound healing experiments in 5-8F (**c**) and 6-10B (**d**) cells. The invasion of different cell groups was detected by transwell invasion assays in 5-8F (**e**) and 6-10B (**f**) cells. The results represent the average of three independent experiments ± standard deviation (S.D). (**P < 0.01)

To investigate the role of miR-324-3p on NPC migration and invasion, we upregulated the expression of miR-324-3p by transfecting miR-324-3p mimic into NPC 5-8F and 6-10B cells. A transfection efficiency of 93.8 ± 2.1 and 92.7 ± 3.3% was observed under fluorescence microscopy in 5-8F and 6-10B cells, respectively, and the expression of miR-324-3p was successfully increased (*P* < 0.01; Fig. 1a, b). The wound healing assay showed that cell migratory abilities of miR-324-3p overexpressing cells were greatly inhibited compared with uninfected mock and control cells (*P* < 0.01, Fig. 1c, d). The transwell invasion assay showed that the number of invading cells was significantly decreased in miR-324-3p upregulated cells (*P* < 0.01, Fig. 1e, f). These results indicated that ectopic expression of miR-324-3p could inhibit migration and invasion of NPC cells.

### Silencing expression of WNT2B suppresses migration and invasion of NPC cells

**Fig. 2:**
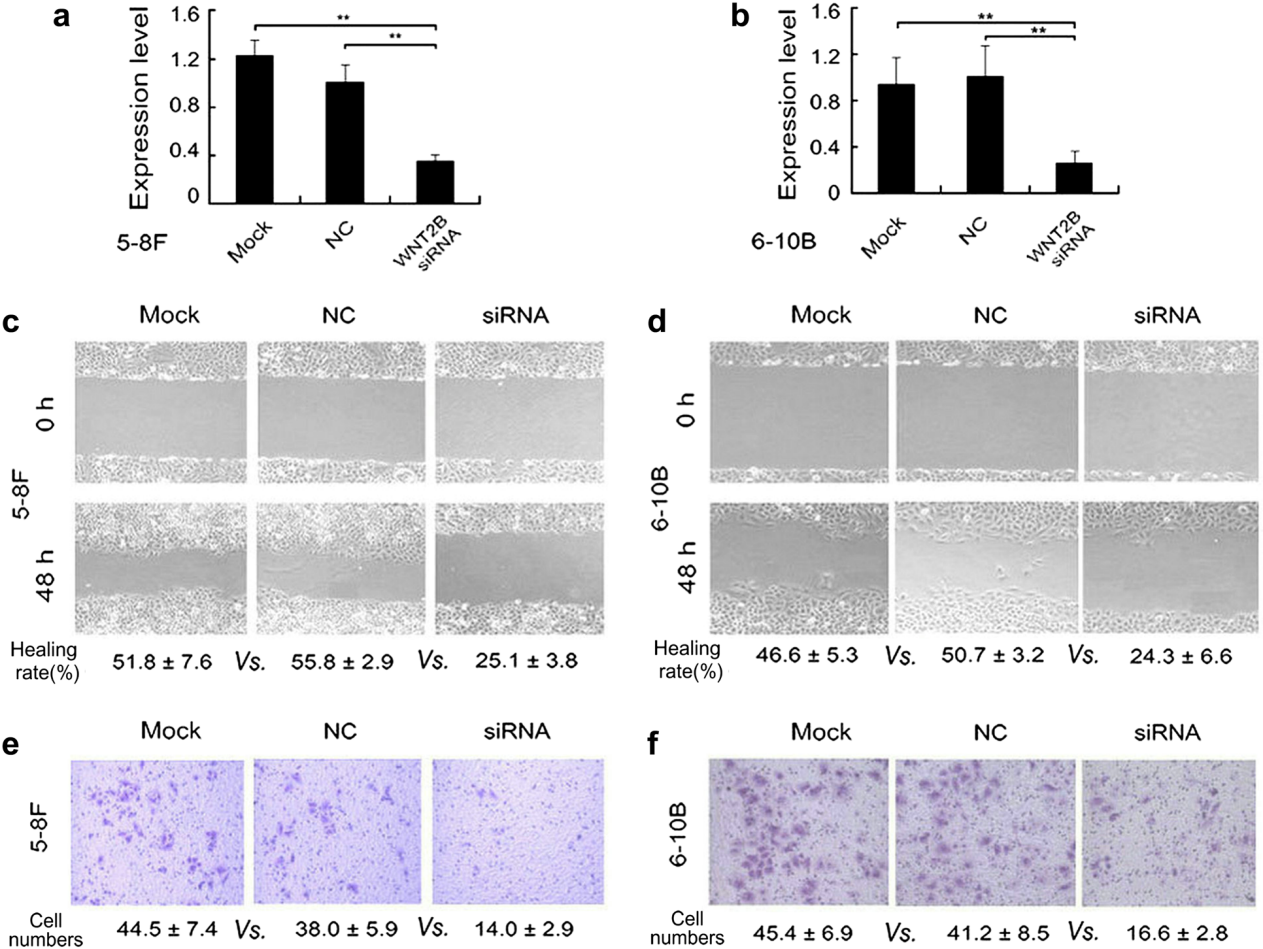
Silencing expression of WNT2B suppresses migration and invasion of NPC cells. WNT2B was successfully downregulated by WNT2B siRNA in 5-8F (**a**) and 6-10B (**b**) cells. The migration of different cell groups was examined by wound healing experiments in 5-8F (**c**) and 6-10B (**d**) cells. The invasion of different cell groups was examined using matrigel invasion assays in 5-8F (**e**) and 6-10B (**f**) cells. The results represent the average of three independent experiments ± standard deviation (S.D). (**P < 0.01)

Our previous study had shown that WNT2B was the target gene of miR-324-3p, so we transfected WNT2B siRNA plasmids into NPC cells to confirm its role on migration and invasion of NPC. The expression of WNT2B was obviously decreased in 5-8F and 6-10B cells by qRT-PCR detection (Fig. 2a, b). The wound healing assay demonstrated that cell migration ability was inhibited when WNT2B was downregulated (*P* < 0.01, Fig. 2c, d). The transwell invasion assay showed that the number of invading cells was significantly reduced in WNT2B siRNA transfected cells (*P* < 0.01, Fig. 2e, f). These data confirmed that the target gene WNT2B could also affect the migration and invasion of NPC cells.

### miR-324-3p alters the expression of EMT biomarkers E-cadherin and Vimentin

**Fig. 3:**
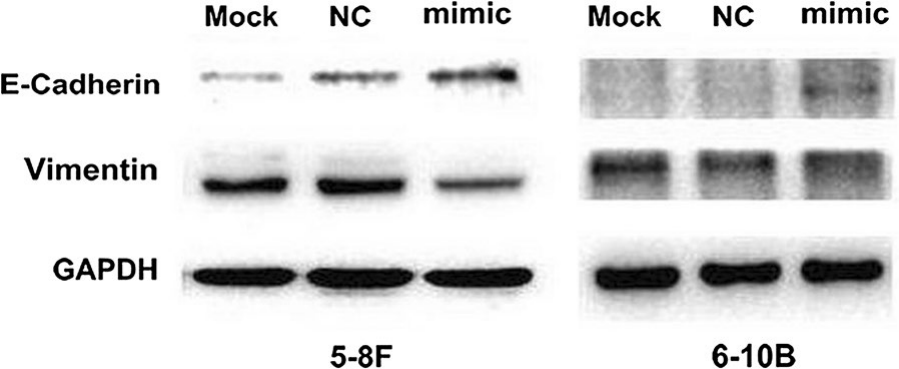
miR-324-3p alters the expression of EMT biomarkers E-cadherin and Vimentin. Following ectopic expression of miR-324-3p, the expression of E-cadherin was upregulated and Vimentin was downgrelated, shown by Western blotting

EMT was a classical mechanism of tumour invasion and metastasis, and we had confirmed that WNT2B could affect the expression of EMT biomarkers in previous study [14]. Here, we found following the upregulation of miR-324-3p in both NPC 5-8F and 6-10B cells, the expression of epithelial markers E-cadherin was upregulated while mesenchymal markers Vimentin was downregulated (Fig. 3), which suggested that EMT might participate in the process of miR-324-3p mediated migration and invasion of NPC.

### miR-324-3p is downregulated and its target gene WNT2B is upregulated in NPC specimens

**Fig. 4:**
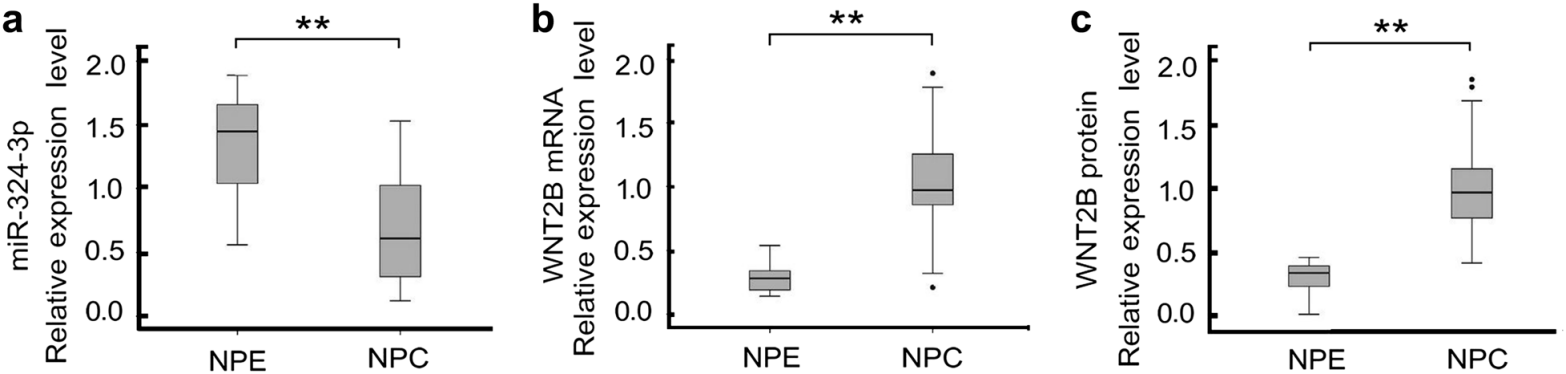
The expression of miR-324-3p and WNT2B in NPC specimens. **a** miR-324-3p was downregulated in NPC tissues compared with NPE tissues. **b** WNT2B mRNA was upregulated in NPC tissues compared with NPE tissues. **c** WNT2B protein was upregulated in NPC tissues compared with NPE tissues. miR-324-3p, WNT2B mRNA and protein were normalized with the internal control U6, GAPDH and β-Actin, respectively. (**P < 0.01)

We have validated that both miR-324-3p and target gene WNT2B could affect migration and invasion of NPC, so we further quantified the expressions of miR-324-3p and WNT2B in 39 freshly frozen NPC and 21 normal NPE tissues. The results showed that in NPC tissues, the expression of miR-324-3p was significantly decreased and both WNT2B mRNA and protein were upregulated (Fig. 4). And the expression of miR-324-3p was negative correlated with WNT2B mRNA and protein (Additional file 2: Figure S3).

### Correlationship between miR-324-3p/WNT2B expression and clinicopathological parameters

The relationship between miR-324-3p/WNT2B expression and clinicopathological characteristics of NPC was explored. As summarized in Table 1, miR-324-3p lower expression was associated with tumour T classification, clinic stage and lymph node metastasis (*P* < 0.05), respectively. And WNT2B mRNA/protein overexpression was also associated with tumour T classification, clinic stage and lymph node metastasis (*P* < 0.05). However, no significant relationship existed between these markers and variables such as gender and age (*P* > 0.05). More expression data of NPE and NPC with two differential stratifications of T and clinic stage forms was shown in Additional file 2: Figure S4.

**Table 1:** Clinicopathological features of the cases of NPC

Variables	Cases	miRNA-324-3p	WNT2B mRNA	WNT2B protein
Sex				
Male	28	0.717 ± 0.463	1.028 ± 0.461	1.001 ± 0.369
Female	11	0.613 ± 0.245	1.052 ± 0.305	1.010 ± 0.320
Age				
<45	19	0.731 ± 0.386	1.055 ± 0.372	1.008 ± 0.301
≥45	20	0.647 ± 0.443	1.016 ± 0.468	0.999 ± 0.402
T stage				
T3 + T4	9	0.476 ± 0.296	1.306 ± 0.324	1.312 ± 0.340
T1 + T2	30	0.751 ± 0.426*	0.953 ± 0.414*	0.911 ± 0.303**
Clinic stage				
III + IV	23	0.466 ± 0.283	1.237 ± 0.341	1.199 ± 0.304
I + II	16	1.006 ± 0.362**	0.743 ± 0.347**	0.722 ± 0.187**
Lymph node metastasis				
Yes	28	0.539 ± 0.324	1.171 ± 0.316	1.099 ± 0.284
No	11	1.067 ± 0.381**	0.687 ± 0.461**	0.761 ± 0.403*

Data was shown as mean ± SD. miR-324-3p, WNT2B mRNA and protein were normalized with the internal control U6, GAPDH and β-Actin, respectively

* *P* < 0.05

** *P* < 0.01

## Discussion

To understand the underlying molecular mechanisms involved in NPC migration and invasion is beneficial to develop better therapeutic strategies for NPC patients. Recently, as important regulatory factors, miRNAs have been shown to play crucial roles in various malignant biobehaviours of tumours, including NPC migration and invasion. Our previous study indicated that miR-324-3p could target *WNT2B* to affect the radioresistance of NPC in vitro [13]. In this study, we further demonstrated that miR-324-3p could also regulate the migration and invasion of NPC, which contributes to illustrate the complex molecular mechanisms of migration and invasion in NPC.

miR-324-3p was located in the region of human chromosome 17p13.1 and firstly identified in mammalian neurons [15]. Up to now, with the rapid development of microarray and sequencing technology, some researchers have found that miR-324-3p was dysregulated in a variety of tumours such as breast cancer [16], hepatocellular carcinoma [17] and pancreatic cancer [18]. However, none of these studies further explored the roles of miR-324-3p on tumour maglinant biobehaviours. More specifically, previous investigations into the function of miR-324-3p were limited, and only several reports showed miR-324-3p was a target of ACE inhibition to promote renal fibrosis and miR-324-3p could target RelA promoter to induce its expression in an Ago2 dependent manner in cells of neural origin [19, 20]. Functional analyses of miR-324-3p on tumour were just seen in our preceding study [13] and another similar report of NPC radioresistance, in which *SMAD7* was validated as the target of miR-324-3p [21]. In this study, we further confirmed the role of miR-324-3p on NPC migration and invasion, which suggests miR-324-3p as an anti-tumour miRNA.

miRNAs usually exert their function by interacting with their target genes via base pairing. In our previous research, we found that *WNT2B* was a direct target gene of miR-324-3p and confirmed *WNT2B* could affect radioresistance of NPC cells [13]. *WNT2B* was known to stimulate the canonical WNT/β-catenin pathway and affected various malignant tumour progression [22]. In head and neck squamous cell carcinoma, *WNT2B* played a role in tumourigenesis and chemotherapy resistance in vivo and in vitro [23]. Intratumoural *WNT2B* was reported to be correlated with the expression of Survivin and c-Myc, tumour proliferation and prognosis in malignant pleural mesothelioma [24]. Jiang et al. also found that the high-expression level of *WNT2B* was associated with the progression and worse outcome of pancreatic cancer [25]. In addition, *WNT2B* can increase the ability of metastasis and chemoresistance of ovarian cancer through the caspase-9/BCL2/BCL-xL pathway and EMT/p-AKT pathways [26]. All these studies highlighted the role of *WNT2B* on tumour malignant processes. However, the function of *WNT2B* on NPC was only seen in our preliminary work, and here we further found that *WNT2B* played roles on migration and invasion of NPC, and revealed that both *WNT2B* mRNA and protein was positively correlated with higher stages of NPC, which indicated the possibility of *WNT2B* to be the novel biomarker of NPC.

EMT was defined as a process that epithelial cells transformed into a mesenchymal phenotype, in which the expression of epithelial marker E-cadherin and mesenchymal marker Vimentin was considered as the classical indicators [27]. And EMT had been widely investigated to be classical mechanism of tumour invasion and metastasis [27, 28]. Moreover, recent researchers reported that miR-544a could induce EMT through the activation of WNT signaling pathway in gastric cancer [29]. In other tumours such as ovarian [30], colorectal [31] and tongue cancer [32], EMT was also associated with the activation of the WNT signalling pathway. With regard to NPC, we found that *WNT2B* was able to change the expression of E-cadherin and Vimentin [14]. In this study, we validated that miR-324-3p also had the ability of altering the expression of EMT biomarkers, which showed that miR-324-3p might regulate the migration and invasion of NPC through EMT.

In summary, we have demonstrated that miR-324-3p can target *WNT2B* to regulate migration and invasion in NPC, and both miR-324-3p and target gene *WNT2B* were associated with T stage, clinic stage and cervical lymph node metastasis. Therefore, miR-324-3p/WNT2B axis may be potential therapeutic target for the treatment of patients with NPC.

## Conclusion

miR-324-3p is a tumor-suppressive miRNA in NPC and inhibit NPC cell migration and invasion by targeting *WNT2B*. Complete understanding of the miR-324-3p/*WNT2B* pathway might contribute to discover new potential therapeutic clues for NPC.

## Additional files

**Additional file 1.** qRT-PCR technical documentation.

**Additional file 2: Figure S1.** Representative data of standard and melt curves of the premirs. (A) Standard curves of GAPDH and WNT2B. (B) Melt curves of GAPDH. (C) Melt curves of WNT2B. (D) Standard curves of U6 and miR-324-3p. (E) Melt curves of U6. (F) Melt curves of miR-324-3p. **Figure S2**. Validation of the stability of the reference genes. (A) Amplification plot and CT values of U6 between NPC and NPE. (B) Amplification plot and CT values of GAPDH between NPC and NPE. **Figure S3.** Spearman correlation analysis between the expression of miR-324-3p and WNT2B. (A) miR-324-3p was negative correlated with WNT2B mRNA. (B) miR-324-3p was negative correlated with WNT2B protein. **Figure S4.** The expression data of NPE and NPC with two differential stratifications of T and clinic stage forms. (A) The expression of miR-324-3p between NPE and NPC. (B) The expression of WNT2B mRNA between NPE and NPC. (C) The expression of WNT2B mRNA between NPE and NPC.

## Abbreviations

NPC: nasopharyngeal carcinoma
NPE: nasopharyngeal epithelium
NC: negative control
EMT: epithelial-mesenchymal transition
UTR: untranslated regions
qRT-PCR: quantitative real time PCR
