# Supplementary material for: miR-324-3p suppresses migration and invasion by targeting WNT2B in nasopharyngeal carcinoma
Source: Cancer Cell Int. 2017 Jan 3;17:2. doi: 10.1186/s12935-016-0372-8 (PMC5209830; doi:10.1186/s12935-016-0372-8)
Supplement: Supplementary file 1 — Additional file 1. qRT-PCR technical documentation. [file 12935_2016_372_MOESM1_ESM.docx]

**Additional file 1. qRT-PCR technical documentation.**

Reverse transcription of miRNA (GeneCopoeia Inc., MD, USA, Cat. No. AOMD-Q060)

| Reagent | Volume |
| --- | --- |
| Total RNA | 2 μl (1 μg) |
| 2.5 U/µl Poly A Polymerase | 1 μl |
| RTase Mix | 1 µl |
| 5×PAP/RT Buffer | 5 µl |
| dd H_2_O | 16 µl |
| Final Volume | 25 µl |
| Incubate at 37°C for 60 minutes and 85°C for 5 minutes | |

Detection of miRNA with qPCR (GeneCopoeia Inc., MD, USA, Cat. No. AOMD-Q060)

| Reagent | Volume |
| --- | --- |
| 2×All-in-One qPCR Mix | 10 μl |
| miR-324-3p/U6 Primer (2 µM) | 2 μl |
| Universal Adaptor PCR Primer (2 µM) | 2 µl |
| cDNA | 2 µl |
| ROX Reference Dye | 0.4 µl |
| dd H_2_O | 3.6 µl |
| Final Volume | 20 µl |

Method for the miRNA qPCR reaction

| Cycles | Steps | Temperature | Time |
| --- | --- | --- | --- |
| 1 | Initial denaturation | 95°C | 10 min |
| 40 | Denaturation | 95°C | 15 s |
|  | Annealing | 62.5°C | 40 s |
|  | Extension | 72°C | 15 s |

Reverse transcription of mRNA (Applied Biosystems, CA, USA, Cat. No. N8080234)

| Reagent | Volume |
| --- | --- |
| 10X RT Buffer | 2 μl |
| 25mM MgCl_2_ | 1.4 µl |
| 10mM dNTP Mixture | 4 µl |
| RNase Inhibitor (20U/µL)  Reverse Transcriptase (50U/µL)  Random Hexamers (50 µM)  Total RNA | 1 µl  1 µl  1 µl  1 μl (0.5 μg) |
| dd H_2_O | 8.6 μl |
| Final Volume | 20 µl |
| Incubate at 25°C for 10 minutes, 37°C for 30 minutes, 95°C for 5 minutes, and 4°C for indefinitely | |

Detection of mRNA with qPCR (Applied Biosystems, CA, USA, Cat. No. 4309155)

| Reagent | Volume |
| --- | --- |
| 2×SYBR Green PCR Master Mix | 10 μl |
| Forward Primer (10 µM) | 1 μl |
| Reverse Primer (10 µM) | 1 µl |
| cDNA | 2 µl |
| dd H_2_O | 6 µl |
| Final Volume | 20 µl |

Method for the mRNA qPCR reaction

| Cycles | Steps | Temperature | Time |
| --- | --- | --- | --- |
| 1 | Initial denaturation | 95°C | 10 min |
| 40 | Denaturation | 95°C | 15 s |
|  | Annealing/Extension | 60°C | 60 s |
