# Supplementary material for: miR-324-3p suppresses migration and invasion by targeting WNT2B in nasopharyngeal carcinoma
Source: Cancer Cell Int. 2017 Jan 3;17:2. doi: 10.1186/s12935-016-0372-8 (PMC5209830; doi:10.1186/s12935-016-0372-8)
Supplement: Supplementary file 2 — Additional file 2: Figure S1. Representative data of standard and melt curves of the premirs. (A) Standard curves of GAPDH and WNT2B. (B) Melt curves of GAPDH. (C) Melt curves of WNT2B. (D) Standard curves of U6 and miR-324-3p. (E) Melt curves of U6. (F) Melt curves of miR-324-3p. Figure S2. Validation of the stability of the reference genes. (A) Amplification plot and CT values of U6 between NPC and NPE. (B) Amplification plot and CT values of GAPDH between NPC and NPE. Figure S3. Spearman correlation analysis between the expression of miR-324-3p and WNT2B. (A) miR-324-3p was negative correlated with WNT2B mRNA. (B) miR-324-3p was negative correlated with WNT2B protein. Figure S4. The expression data of NPE and NPC with two differential stratifications of T and clinic stage forms. (A) The expression of miR-324-3p between NPE and NPC. (B) The expression of WNT2B mRNA between NPE and NPC. (C) The expression of WNT2B mRNA between NPE and NPC. [file 12935_2016_372_MOESM2_ESM.docx]

**Additional file 2**

**Figure S1**


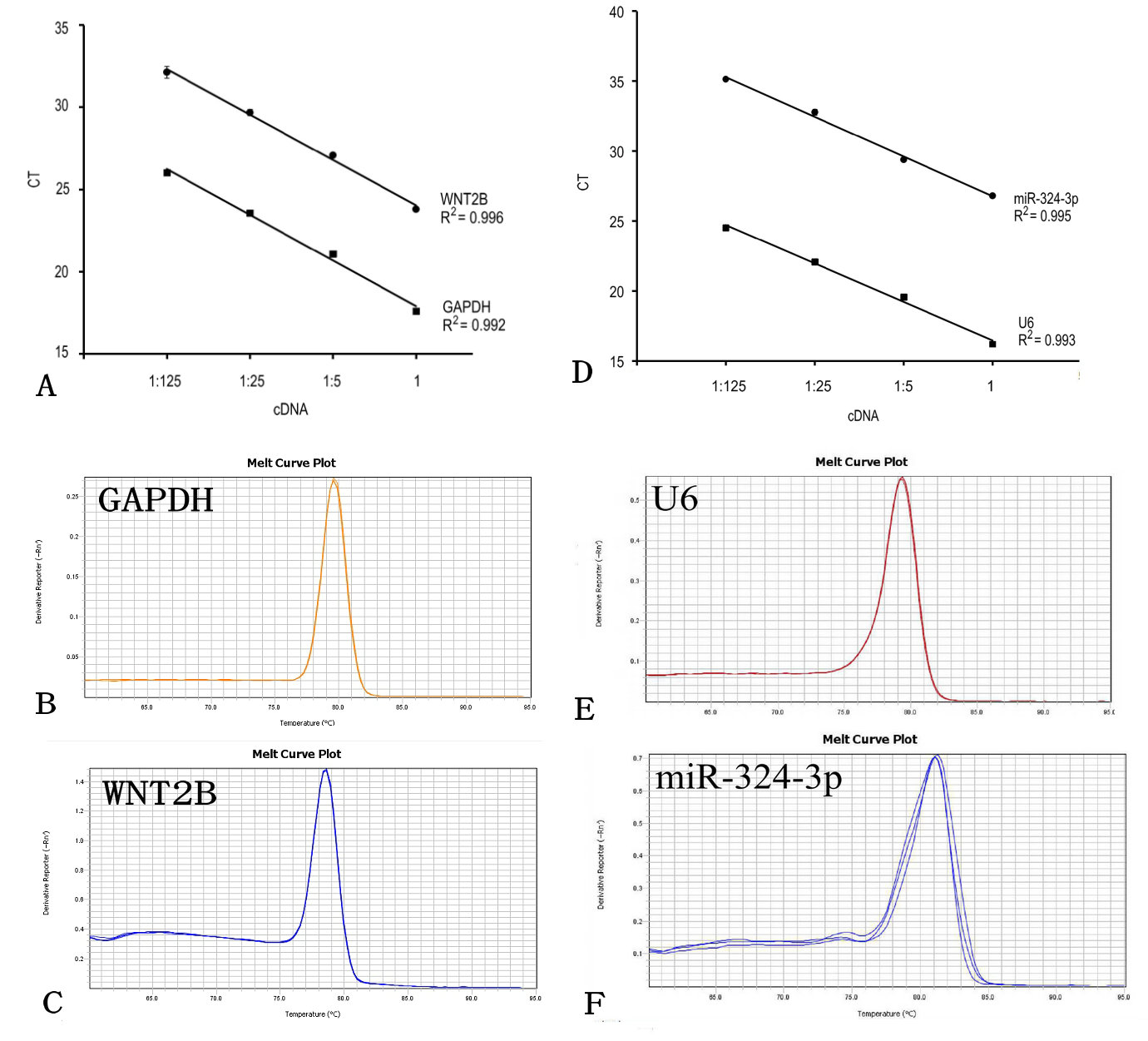


**Figure S1. Representative data of standard and melt curves of the premirs.** (A) Standard curves of GAPDH and WNT2B. (B) Melt curves of GAPDH. (C) Melt curves of WNT2B. (D) Standard curves of U6 and miR-324-3p. (E) Melt curves of U6. (F) Melt curves of miR-324-3p.

**Figure S2**


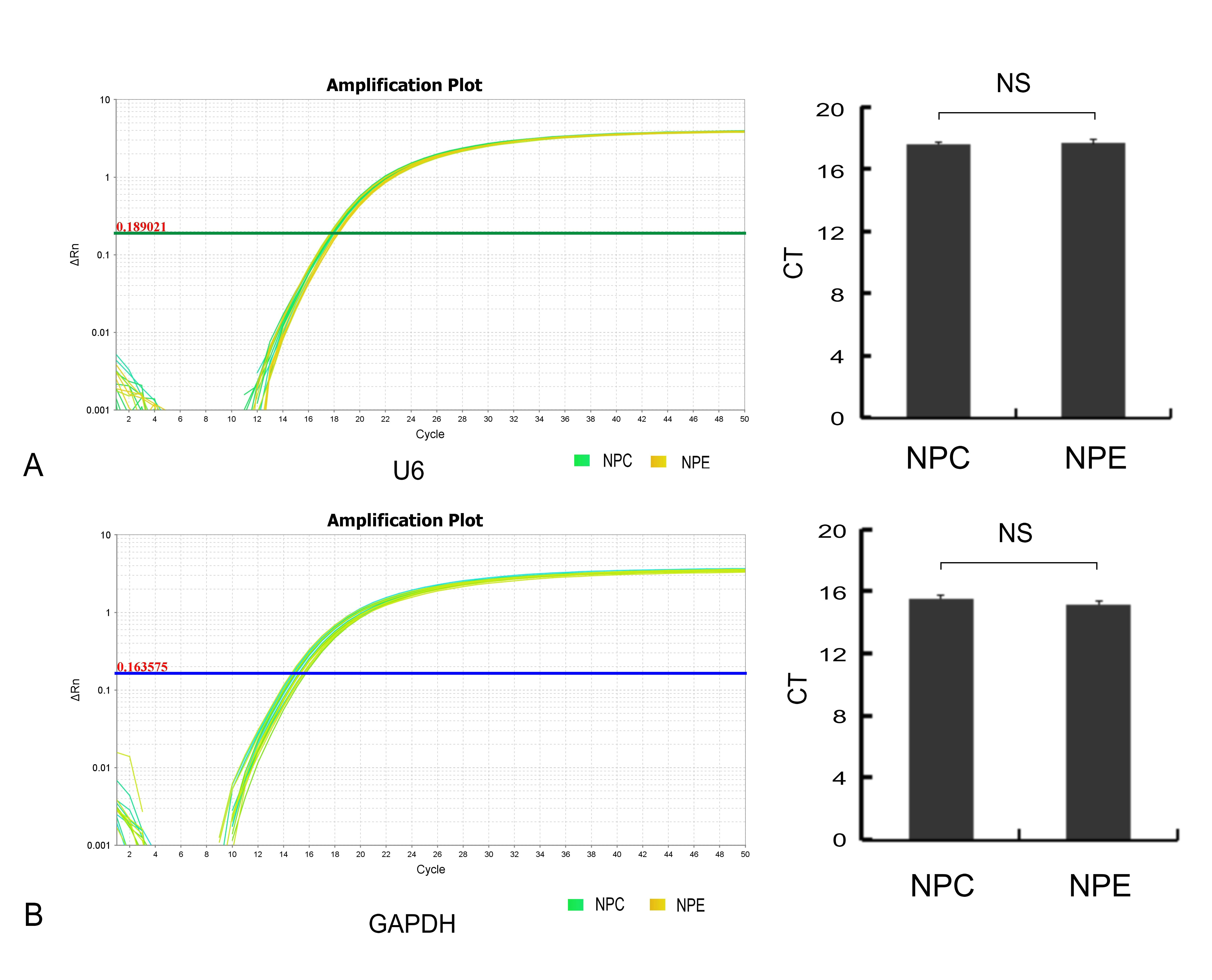


**Figure S2. Validation of the stability of the reference genes.** (A) Amplification plot and CT values of U6 between NPC and NPE . (B) Amplification plot and CT values of GAPDH between NPC and NPE. NS, no significance.

**Figure S3**


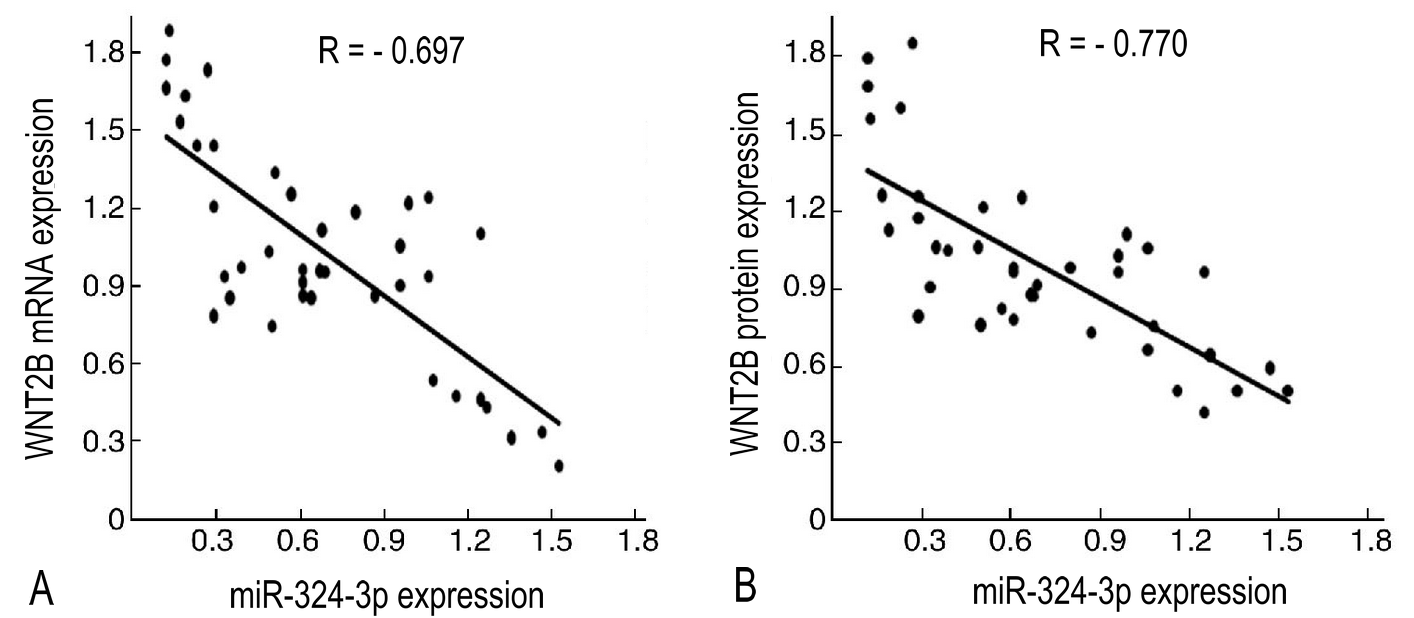


**Figure S3. Spearman correlation analysis between the expression of miR-324-3p and WNT2B.** (A) miR-324-3p was negative correlated with WNT2B mRNA (R = -0.697). (B) miR-324-3p was negative correlated with WNT2B protein (R = -0.770)

**Figure S4**


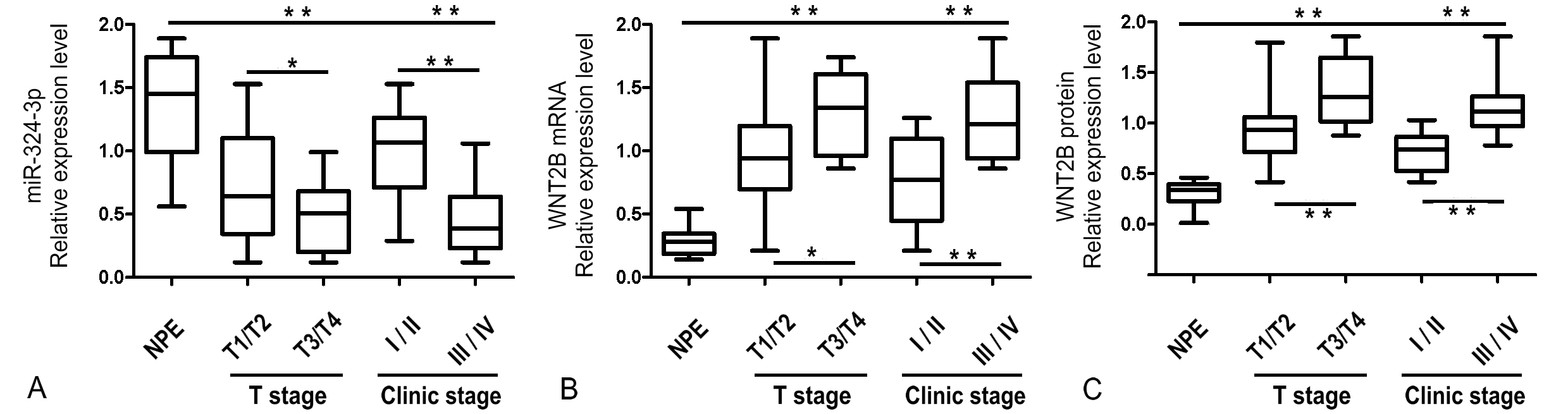


**Figure S4. The expression data of NPE and NPC with two differential stratifications of T and clinic stage forms.** (A) The expression of miR-324-3p between NPE and NPC. (B) The expression of WNT2B mRNA between NPE and NPC. (C) The expression of WNT2B mRNA between NPE and NPC. (* *P* < 0.05, ** *P* < 0.01)
